# Caribbean Heat Threatens Health, Well-being and the Future of Humanity

**DOI:** 10.1093/phe/phv008

**Published:** 2015-04-13

**Authors:** Cheryl C. Macpherson, Muge Akpinar-Elci

**Affiliations:** St George’s University School of Medicine and Windward Islands Research and Education Foundation (WINDREF); Old Dominion University, Center for Global Health, Health Sciences and Windward Islands Research and Education Foundation (WINDREF)

## Abstract

Climate change has substantial impacts on public health and safety, disease risks and the provision of health care, with the poor being particularly disadvantaged. Management of the associated health risks and changing health service requirements requires adequate responses at local levels. Health-care providers are central to these responses. While climate change raises ethical questions about its causes, impacts and social justice, medicine and bioethics typically focus on individual patients and research participants rather than these broader issues. We broaden this focus by examining awareness among health-care providers in the Caribbean region, where geographic and socioeconomic features pose particular vulnerabilities to climate change. In focus groups, Caribbean providers described rises in mosquito-borne, flood-related, heat-related, respiratory and mental illnesses, and attributed these to local impacts of climate change. Their discussions showed that the significance of these impacts differs in different Caribbean nations, raising policy and social justice questions. Bioethics and public health ethics are situated to frame, inform and initiate public and policy dialog about values and scientific evidence associated with climate change. We urge readers to initiate such dialog within their own institutions about the context-dependent nature of the burdens of climate change, and values and policies that permit it to worsen.

‘The better placed an individual is to do what is right, the greater the onus on him to do what is right.’ (Garvey, 2008: 83)

## Introduction

Climate change harms health and well-being. Its consequences, including extreme weather events, cause injuries, exacerbate existing medical conditions, increase exposure to infectious disease through displacement and overcrowding and burden health services. While the World Health Organization, World Bank and similar bodies are investing in research, education and policy aimed at limiting health impacts of climate change that are already accruing (Macpherson, 2013a), bioethics is primarily focused on clinical practice and research. This overshadows attention to broader harms like health impacts of climate change and policies that permit these harms to accrue.

Bioethics ought to loosen its dogmatic focus on autonomy (Dawson, 2010) and instead address issues like how humans are shaped by their natural environments, and how human activities are shaping these environments (Dupras *et al.*, 2014). Moving in this direction, we conducted focus groups with health-care providers about which health impacts of climate change, if any, they had observed in their practices. We conducted our study in the Caribbean region which is particularly vulnerable to climate change due to the geographic and socioeconomic features that define its nations as small island developing states (SIDS). This article frames climate change as a health and bioethics problem, describes our study and the context in which it was conducted and presents our findings. It also discusses the significance and implications of the data for bioethics, and for the future of humanity.

Given the complexity of the problems and the many sectors, disciplines and nations involved, interdisciplinary and nonpartisan deliberation is essential to stabilizing emissions. To advance deliberation, we distinguish between the measurable cause (accumulated atmospheric emissions) and the less measurable impacts (climate change and its diverse and often intangible manifestations). Although each impact damages or destroys resources essential to health and survival, even highly educated individuals concerned about a single impact such as warming may not recognize the consequences of warming for the range of resources or locations affected, recognize other impacts (such as changes to seasonal precipitation) and their respective consequences or differentiate evidence from belief.

## Emissions Matter in Bioethics and Beyond

Climate change refers to decades long and scientifically established changes in regional patterns of wind and precipitation, seasonal weather and average annual temperatures. Its manifestations include extreme weather which causes injuries and displacement and exacerbates chronic conditions like asthma, heart disease and mental illness. Less visibly, it disrupts access to, and availability of, environmental resources that are essential to health and the provision of health care (Macpherson, 2013b); increases exposure to pathogens and disease vectors; and damages the air, water and land upon which we live, play, produce crops and agricultural animals, store goods and provide health care. It also contributes to global warming which, through even fractional increases in average temperatures, expands the ranges and behaviors of some pathogens and disease vectors, and raises sea levels. Sea level rise challenges coastal areas in rich and poor nations, particularly those with high population densities. Two megacities with over 10 million inhabitants in 1950 have since multiplied to about 20 megacities, each generating more pollution and more demand for energy, water, air, food and housing (United Nations, 2010), and creating more challenges for health-care providers, health systems and governments.

Unusual only 10 years ago, extreme weather has become commonplace, causing serious economic and health consequences globally (Jamieson, 2014; Patz *et al.*, 2014). Consequences include the growing risks of extreme heat waves, precipitation and coastal flooding; aggregate economic damages that accelerate with increasing temperature and loss of biodiversity, ecosystems, goods and services; large-scale singular events that abruptly and permanently disrupt physical systems and ecosystems; and uneven distribution of burdens that most harm already disadvantaged individuals and communities (IPCC, 2014a). The burdens include (i) injuries and exacerbation of existing medical conditions, including mental illness; (ii) displacement leading to overcrowding and exposure to infectious and vector-borne disease; (iii) release of chemicals, sewage and pollutants that contaminate food and water; (iv) reduced access to unpolluted fresh water; (v) diminished agricultural productivity and access to food; (vi) disruption of communication, transportation and infrastructure essential to the distribution of supplies; and (vii) greater burdens on health services.

These consequences have attracted relatively little attention in public health ethics, environmental ethics and bioethics, but have prompted a range of work in public health, medicine and beyond. Bioethics and its specializations tend to focus on health and health care for individuals, and to be guided by principles of autonomy, utility and justice, or one of several theories. Because emissions undermine health and the delivery of health care, and raise questions about the extent to which any bioethics principle or theory is fulfilled by responses to emissions or to their consequences in any given setting, one might actually look to bioethics for insight. Bioethics is expert in the analysis and communication of medical risks, and this expertise is equally applicable to the communication of climate risks (Valles, 2015). Bioethics has the potential to inform and drive policies and governance toward mandating substantial reductions in emissions, and by doing this, might help to make health and access to care more equitable.

Many bioethicists recognize that emissions harm health, and that the distribution of related benefits and harms is unfair. Surprisingly few, however, see this as morally problematic, or something they can or should do much about, other than perhaps switching off their computers and lights when not in use. Taking such actions may define us as conscientious citizens but, even collectively, cannot counteract the enormous amounts of emissions generated by industries, institutions and governments; or by globalization that drives demand for products and the energy needed to produce, transport, consume and dispose of them. A rigorous body of work on values and value judgments involved in assessments, policies and practices that allow emissions to rise would help leaders recognize that they themselves, and other stakeholders, will benefit from reducing their emissions. Some bioethicists (including those cited herein and others) are undertaking such work (Deckers, 2011; Dwyer, 2013), but on the whole, it is overdue in bioethics and other sectors, including policy and governance.

To ignite interest among bioethicists and others, we report here what Caribbean health-care providers perceive as impacts of climate change in their respective nations, and discuss the significance of these perceptions. Some of the impacts mirror those reported in other regions by scientists, health professionals and the news media. Paradoxically, while the coastal and low lying geographies of Caribbean nations make them more vulnerable than others to sea level rise and extreme weather, their relatively rapid economic growth is changing consumption patterns in ways that increase global emissions.

## The Study

Building on previous work (Macpherson and Akpinar-Elci, 2013; Maibach *et al.*, 2008) and using a semi-structured approach (Hull *et al.*, 2001), we designed focus group questions to explore perceptions among Caribbean health-care providers including physicians and nurses, and at least one veterinarian and technician in each group. They discussed their professional observations and experiences from within their respective institutions and nations. Only middle- and senior-level participants were included to ensure that they could discuss changes over time. To minimize bias, government officials who might express a ‘party line’ rather than honest perception were excluded, as were public and environmental health experts and others whose knowledge about health impacts of climate change may have overshadowed discussion of their experiences and perceptions.

Institutional Review Board (IRB) approval and informed consent were obtained to audiotape and transcribe discussions. Research coordinators in each nation recruited equal numbers of participants from medicine, nursing, etc, roughly maintaining a gender balance. No inducements were offered. Discussion was conducted in groups of eight in 2013 at times convenient to participants, lasting until the data were saturated (over 2 hours). They were facilitated and audiotaped by C.C.M., observed by M.A.E., minuted by the coordinator and transcribed by a service. Grounded in content analysis, the authors and an external epidemiologist each independently immersed themselves in, and coded, the transcripts, then compared findings to resolve the few discrepancies that arose. For each discrepancy, all analysts had agreed on content but coded it differently. Comments about floods impacting on construction and housing, for example, were coded by two analysts as ‘housing’, and by the third as ‘deforestation’. All data reported here were coded identically by at least two analysts.

## The Caribbean Context

Before presenting findings, we highlight certain Caribbean features to show how health impacts, their local significance and the effectiveness of interventions, vary with location. Clearly, socioeconomic, political and geographic features influence vulnerability, preparedness and willingness and capacity to reduce emissions. Low-income nations, for example, are more severely affected by extreme storms because they lack the construction standards of wealthy nations, have less capacity to prepare and recover and are less able to provide health care to those who are injured or ill.

Geographic factors including coastal, mountainous and desert environments and tropical, temperate and polar climates influence regional flora, fauna, food, clothing, lifestyles and energy use, all of which bear on the significance of specific impacts in those locations. In the Arctic, warming temperatures are melting the permafrost foundation of roads and damaging buildings, causing accidents, and impeding traditional hunting and fishing practices that not only feed indigenous Arctic people and communities but also contribute to their sense of identity (Willox *et al.*, 2012). Warming affects temperate regions differently, contributing in the USA and Russia to severe and prolonged droughts that disrupt seasonal weather patterns and agriculture, reduce access to food and water, and hinder employment, economic growth and security (Associated Press, 2014; Dreibus *et al.*, 2012).

Such changes alter the life cycles and transmission of pathogens and disease vectors that affect humans and other species. Caribbean consequences include the recent appearance or reappearance of mosquito-borne viruses such as malaria and dengue fever in Jamaica, all four dengue serotypes in Grenada (GND) and in 2013, the first cases of Chikungunya (CHKV) in the western hemisphere. Within months, CHKV had infected almost 1000 people in 20 different Caribbean nations (Caribbean Public Health Association, 2014) including over 60% of hospital staff in GND (Beyond the Headlines, Grenada Broadcast Network, 2 September 2014).

## Study Sites: Trinidad and Tobago and Grenada

The Association of Small Island States represents SIDS within the United Nations. About 35 SIDS comprise its membership, including Trinidad and Tobago (T&T), Grenada (GND) and nine other independent English-speaking Caribbean nations with varied socioeconomic, political, geographic, cultural and historic features. Despite their differences, they, like non-Caribbean SIDS, have extensive low lying coastal areas, relatively small percentages of global population, limited global influence, growing reliance on imported goods and disproportionate vulnerabilities to emissions while generating relatively few (SIDSnet, 2014).

Caribbean SIDS have tropical beaches, rain forests, deserts and volcanoes conducive to recreational, creative and inspirational activities that give pleasure, reduce stress and promote health. What remains of their disappearing coral reefs and coastal mangroves and ecosystems still support fishing and tourism, and help buffer effects of storms and sea level rise. These geographies and natural resources generate economic and health benefits in the Caribbean and beyond. These resources and benefits seem less valued than in the past, and how they are valued determines the extent to which they are appreciated, enjoyed and protected, or exploited.

We conducted our study in the larger and more industrial T&T, and the smaller and less developed GND, to explore how the impacts and significance of emissions differ with context. Separated by 100 miles, T&T’s population is predominantly of African and Asian Indian heritage with a small percentage of other backgrounds, and GND’s is primarily of African heritage. Bachelor degree programs have been available for over 50 years in T&T, and under 20 years in GND. World Bank data for 2012 (the most recent available at the time of writing) shows their respective surface areas as 5130 and 340 square kilometers; population sizes as 1,337,439 and 105,483; gross domestic products as US$17,000 and US$7000; health expenditures per capita US$972 and US$478 contrasted with US$8895 in the USA and US$3495 in the UK; and most recent ‘CO2 emissions (metric tons per capita)’ in 2010 as 38.2 and 2.5 contrasted with 7.9 in the UK and 17.6 in the USA, with T&T’s emissions outranked only by Qatar at 40.3 (World Bank Data, 2014).

## The Data

After introducing everyone present, and reminding participants that we did not expect them to have expertise in environmental health or climate change, we invited them to discuss a series of questions from the perspective of their own observations and experiences. The first question, to avoid imparting bias, intentionally omitted the phrase climate change, asked ‘*What environmental changes do you think are occurring that now affect, or will likely affect, health in your nation?*’.

Agreement was strong among both groups that environmental changes are occurring in their respective nations and reducing agricultural productivity; increasing the frequency and magnitude of local landslides, floods and pollution; and affecting health and hospital admissions. Discussions in both groups were animated and generated comments like those below, which were further addressed during subsequent questions.

- ‘Once there’s a flood you know within the next weeks you expect to see people coming in with fever, generalized body pain, rash, and all of a sudden they can’t pass urine … they have pneumonia or some fungal infection.’ (T&T)
- ‘If anyone’s looking at what’s happening in the oceans and with our rivers. In the dry season if you get close to these big industries, when it rains here and the rivers get plugged with the other stench and stuff, and we don’t have an idea of what is in that black weed stuff.’ (T&T)
- ‘If you have a prolonged dry season, then you have 3 or 4 days of heavy rain, the week after that you tend to see a lot of cases of gastroenteritis coming in.’ (GND)
- ‘Definitely the air is more polluted, the quantity of vehicles we have on the road now … We’re even guilty at the hospital—our incinerators—because I have allergies and whenever they burn the stuff I can’t open my eyes.’ (GND)

Based on 10 topics specified in a study of health department preparedness in the USA (Maibach *et al.*, 2008), participants were then asked ‘*What do you think is the significance of the following health impacts in your nation now, and 5 years from now?*’. Responses are highlighted below by topic.

### Heat Waves and Heat-Related Illnesses

Participants in both groups perceived air temperatures as hotter than usual and as contributing to more heat-related illness and hospital admissions. GND participants described an increase in hospitalizations for dehydration and sunburn, while T&T participants talked extensively about heat stress, chronic obstructive pulmonary disease and increasing deaths of working and domestic animals. One said ‘I can’t really tell you for sure if it’s because of the number of dogs in society, or the number of true cases as a result of what’s happening with the temperature in the environment. But we are seeing these dogs and they collapse, they’re suffering from heat stress. We have them on fluids, trying to cool them down to save their lives’.

### Storms Including Hurricanes and Floods

Both groups perceived increases in mosquito-borne disease, diarrhea and flood-related illness, and declines in subsistence agriculture which they described as once widely undertaken by rich, as well as poor, families. The disappearing distinction between dry and wet seasons was raised in T&T during this and subsequent questions, and in GND, during subsequent questions. T&T participants also described increasingly frequent floods affecting increasingly larger areas.

- ‘With 2–3 days of rain we get people dislocated when their homes are destroyed, and the more we clear down our lands to build houses, I think the more we’re going to have flooding with the slightest rain.’
- ‘When the flood comes, they [tethered animals] have no way to escape and they just stay and die. That’s a welfare concern for us as well. And this affects the food supply here. … Every time there is a flood, the price of vegetables goes up.’
- ‘A lot of the flooding relates to indiscriminate land use … but some is definitely due to increases in the amount of rainfall we’ve been getting. … We used to have a distinct dry season or wet season. Now that no longer exists, we have rain anytime. … If we get one heavy shower or rainfall, we get flooding.’

After 49 years without a hurricane, GND suffered major damage from Hurricane Ivan in 2004. GND participants described overcrowding and depression that persisted even a year later, and expressed concern that if a similar event occurred today, there would be less foreign aid and investment in GND’s recovery due to the global economic downturn.

- ‘If you have a house equipped to hold 5 people, you have 20 now. So when 1 person gets sick, everybody gets sick.’
- ‘We’re still recovering. Our nutmeg was wiped out, the animal population declined, and these are all just starting to pick up again.’
- ‘The world economy was a lot better then. We got a lot of help [foreign aid], but right now we won’t be able to get all this help.’
- ‘If something like Ivan was to happen again, persons are going to be even more depressed than before.’

### Drought, Forest Fires or Brush Fires

Both groups described ‘bush fires’ as more frequent and visible, and the burning of household and other waste as widespread. GND participants said that bush fires are illegal but are used anyway. Lack of resources or official willingness with which to challenge traditional or widespread practices like this may occur in many locations, and may also impede efforts to limit emissions.

T&T participants described droughts as becoming more frequent due to changing seasonal patterns, and described how droughts worsen floods and affect agricultural animals. One said ‘In the dry season when there’s limited water supply, birds [poultry] would just die. They need water all the time’. Another said ‘The undergrowth doesn’t seem to be as dry. Rain comes and dampens things and we don’t have the extensive wild fires that we would have had lets say 3 years ago … If we go back to very long dry spells I’m fairly certain a return to those serious wild fires would have consequences for flooding’.

### Vector-Borne Infectious Diseases

Both groups perceived mosquito-borne disease as increasing, and attributed this to changing seasonal patterns that affect mosquito breeding and biting.

- ‘As we lose the distinction between dry and wet seasons, we may have an early mosquito season. That would contribute to more dengue, more yellow fever, malaria. … And if we have more flooding, more water-borne disease, cholera, salmonella.’ (T&T)
- ‘We’re not having seasons anymore—you don’t see the clear cut rains and clear cut dry.’ (GND)

### Anxiety, Depression or other Mental Health Conditions

Both groups perceived increases in mental illness and expressed concern that stigma hinders willingness to seek treatment. Attempted suicides were perceived as increasing in GND but not linked directly with climate change. T&T participants agreed that ‘A lot of it [mental illness] is stress-related and increasing, and whether the increasing is a result of environmental changes or the greater stress of modern living we really don’t know’. One said that rising rates of depression affect ‘the way in which we manage patients. Because they don’t have the zeal, they are not compliant’.

### Quality or Quantity of Fresh Water Available

Both groups described a growing reliance on bottled water, and the quality of tap water as poor during floods and the dry season. In T&T, tap water has ‘a bitter aftertaste’ and ‘a white cloudy foully stench sometimes, and sometimes it’s so strong … in some areas you cannot drink tap water now’. Several attributed this to pollution, and one to poorly dissolved water purification chemicals. GND participants described some of their rivers as drying up, and described their water quality as good ‘most of the time’ but water storage and distribution systems as needing strengthening. GND participants said:

- ‘They should be looking at more storage facilities because during the dry season we do have severe problems.’
- ‘When it’s not good you know it. When it floods, it comes dirty. You can see and so you don’t drink it.’
- ‘Years ago if you thirsty you could stop at a pipe and drink. Now everybody has their little water bottle.’

### Unsafe or Ineffective Sewage and Septic System Operation

T&T participants said that floods cause septic tank leakage and contamination that lead to infections requiring medical care. Reflecting GND’s economic reliance on fishing and tourism, participants focused on damages to their beaches and fish from sewage being pumped out to sea, saying:

- ‘Well what is it [sewage] being treated with and how would that affect us in the long run?’
- ‘A lot of our corals are dying.’
- ‘Some of the fish, the bottom feeders, are not safe to eat, barracuda and things like that.’

### Food Safety and Security

Both groups discussed growing reliance on imported foods. In T&T, one said ‘Trinidad has never been a country that strives to be food sufficient in terms of our own production, just because of the belief we have oil so we can buy foods’. T&T participants also said that farming is being disrupted by construction on what was formerly agricultural land, and by the pursuit of education, urban lifestyles and higher salaries. They talked extensively about crop damage due to heavy rains and changing weather patterns, for example, the ‘problem with tomato production now … the rain is going to beat the flowers off and we’re seeing it from crop to crop because of the changes in our weather pattern’.

GND participants said that local farming incomes would improve if farmers moved into organics and improved presentation of their produce. One said that a Caribbean Community workshop on food security had recently been held in Grenada. GND’s comments (below) resemble those from T&T.

- ‘What was considered agricultural land has now become commercial land.’
- ‘All imported foods are cheaper than the local.’
- ‘They import the feed for the cattle, chicks, all these things.’
- ‘I think in the past we did a lot more farming. Actually people keep small farms and this is something that is pretty much in the past. Now no one wants to do that. Rather than plant a few tomatoes and so, you go, you buy it in the supermarket and its right there.’
- ‘We have to be able to take care of ourselves and we are going to starve if something really was going to happen worldwide. … We have a supermarket, great, were happy about it. But if something was to happen we can’t sustain our selves.’

### Housing for Residents Displaced by Extreme Weather Events

T&T participants described the cost of housing therein as very high, and as a significant source of stress, even to the wealthy, anticipating that this stress will worsen as extreme weather increases demand for housing. GND participants described construction standards as having improved, and emergency shelters as more available, since Hurricane Ivan.

### Health-Care Services for People with Chronic Conditions during Service Disruptions such as Extreme Weather Events

T&T participants observed growing preparedness within their health-care system but were uncertain about readiness.

- ‘I find in normal times we can’t supply the needs of the public. For instance with the dialysis, the people who need dialysis in this country on a regular basis—no [we are unprepared].’
- ‘They have upgraded the health centers to have mini-operating theatres … we’re trying to extend the hours gradually, and the reason for doing that is to put less pressure on the main hospital … I’ll say our doctors are ready, our staff is ready for a major disaster. The government has the money but … the gauze and the plasters and all these things—do we have it in stock?’

GND participants instead discussed the vulnerability of Grenada’s main hospital which is coastally located and has limited road access, and expressed concern that stakeholders lack information.

- ‘We have a lot of connecting roads in Grenada that people use, but they are not equipped to handle the heavy traffic where a major road is disrupted.’
- ‘I would hear “we’re doing this” and “we have this plan in place.” Truthfully the doctors and nurses right on the ground don’t know anything about that.’

## Significance of the Data

### Similarities, Differences and Generalizability

That health-care providers in the Caribbean perceive emissions and climate change as impacting on their patients now highlights the need to understand emissions as a present, as well as future, problem. It also shows that providers are confronted with managing the health risks and changing health service requirements resulting from the complex impacts of droughts, rain, floods and hurricanes. Responses common to both nations include (i) increasing heat- and respiratory-related illness due to rising temperatures; (ii) increasing mosquito-borne and flood-related illness due to changing seasonal weather patterns; (iii) poor water quality during floods and the dry season; (iv) more mental illness deriving from greater stress in society; and (v) declining agricultural productivity and food security as reliance on bottled water and imported food increases. That scientific and news media reports have documented some of these problems in other locations suggests that they are generalizable within and beyond Caribbean SIDS. For ease of discussion, Table 1 summarizes and contrasts the impacts described in T&T and GND and contrasts these with the differences between them.

**Table 1.** ‘What do you think is the significance of the following health impacts in your nation now, and 5 years from now?’

	Both	T&T	GND
Heat waves and heat-related illnesses	Increased Admissions for *Heat stroke* *Heart attack*	Increased Heat stress (human and animal) COPD	Increased Dehydration Sunburn
Storms (including hurricanes and floods)	Increased Flooding Diarrhea Mosquitoes Mosquito-borne disease	Increased Animal death Agricultural loss Dengue fever Yellow fever Decreased Water quality (and related disease)	Increased Depression (especially elderly and uninsured) Crowding Disease clusters
Droughts, forest fires or brush fires	Increased Bush fires	Increased Harms involving *Livestock* *Crops* *Water distribution* *Floods* *Seasonal patterns*	Ignore fire bans
Vector-borne infectious diseases	Increased Mosquitoes Dengue fever	Increased Cholera Yellow fever Zoonotic diseases *H1N1* *Rabies* *Rat aggression*	Poor mosquito control
Anxiety, depression or other mental health conditions	Increased Depression	Increased Mental illness	Increased Suicide attempts
Quality or quantity of fresh water available	Increased Use of bottled water Decreased Water quality in floods and dry season	Questionable quality Rusty pipes Inadequate supply	Rivers drying up Inadequate storage
Unsafe or ineffective sewage and septic system operation		Septic tanks leak (especially in floods)	Harms fish and coralsHas improved
Food safety and security	Increased Imported food use		Decreased Farming
Housing for residents displaced by extreme weather events		Increased Stress House prices	Improved Construction quality Number of shelters
Health-care services for people with chronic conditions during service disruptions such as extreme weather events		Increased Preparedness Upgrades to health centres	Vulnerable location of main hospital

The differences are important because they reflect the different contextual features of each. GND is economically reliant on fishing and tourism, for example, and the beaches lining much of its coastline are enjoyed by its people, many of whom live and work in close proximity. GND participants thus focused more on the sea and coastal regions than T&T participants. Experiences of Hurricane Ivan and its aftermath grounded observations in GND regarding depression, infectious disease and anxiety that it would receive less international assistance if another hurricane were to strike. Issues raised by T&T participants emphasized other features. They expressed more concern about floods which they repeatedly said occur more frequently, affect larger areas and cause greater damage and illness than in the past. They attributed this to changing seasonal weather patterns that compound the effects of clearing and construction on previously undeveloped land and hillsides. While GND participants also discussed changing weather patterns, they said relatively little about clearing of, or construction on, land. T&T’s discussions about air and water pollution are consistent with its oil extraction and refineries, and factories producing detergents and fertilizers. Despite contextual similarities between T&T and GND, the differences described here demonstrate that local context bears on the types and significance of the impacts of emissions, and suggest that causes and effective responses will also differ with context.

### Implications

For responses to be effective, development and aid programs must be sensitive to local socioeconomic, cultural and environmental conditions, and designed to empower communities and optimize environmental benefits of their traditional practices (IPCC, 2014b). There is limited commitment to providing such programs, and limited capacity in low- and middle-income nations for negotiating its provision, but bioethicists could inform and initiate dialog in SIDS and other nations about the value of implementing local and regional policies that mandate environmentally friendly construction standards or energy-saving materials and designs. Doing so could help build host nation capacity to negotiate, for example, such standards into hotel and business development deals. Given global demand for, and depletion of, sand used in cement production (Gillis, 2014), such capacity could have particular relevance in SIDS and other low- and middle-income countries (LMIC) with sandy beaches. Aid, development and investment programs that use public goods like undeveloped beachfronts, mangroves, forests, rivers and hillsides ought to be designed to help prevent or offset harms to these natural environments, particularly when disproportionately large economic benefits are involved.

Globalization can boost employment and economic growth but also raises massive corporate profits, and global emissions. While development through globalization can improve some socioeconomic and health indicators, policies that facilitate globalization by marketing Western products and lifestyles in LMIC, and raising global consumption and emissions, counteract the benefits. In his review of economic debates and models, Dale Jamieson (2014) shows that economic forecasts and risk assessments do not assign realistic monetary values to the environmental resources and weather patterns on which industrial and national productivity depend, or incorporate the costs to industries or host nations of damaging these resources. This failure undermines existing economic arguments about emissions, and along with lack of information and insight, underlies widespread acceptance of policies that permit emissions to rise.

The balance between economic and health benefits and burdens warrants reexamination. Where the balance has turned, policies, practices and priorities must be modified because, as economist Juliet Schor (2010: 22) explains, what ‘was efficient for constructing the nineteenth-century industrial economy is not what’s most suited for the resource-scarce system of the twenty-first’. Bioethicists, like other professionals, have opportunity and influence with which to alter emissions-related policies. They can elucidate why patients, providers and others should care about, and work to reduce, emissions in medical specialties such as assisted reproduction (Richie, 2014); and explore how to reduce depression, speed recovery, improve patient outcomes, have a calming effect on staff, save money and conserve energy in hospitals by, for example, adopting evidence-based designs like larger windows and easily accessible public gardens (Herbert, 2011; Sadler *et al.*, 2011; Yordy, 2011).

The concept of global bioethics put forth by Van Rensselaer Potter enthusiastically promotes actions and policies likely to benefit the health of present and future generations, underprivileged and vulnerable populations and natural environments (Dupras *et al.*, 2014; ten Have, 2012). We use this bioethics to frame our findings in the hope of catalyzing interdisciplinary partnerships aimed at illuminating connections between health and nature, and encouraging stewardship of natural environments for their extrinsic value to health and well-being, and for their intrinsic value.

## Bioethics has a Responsibility to Help

### Advancing Justice

With exceptions like T&T, LIMC produce fewer emissions than wealthy nations but suffer greater harms, have less capacity than others to recover and receive fewer benefits. Jamieson (2008) documents some of the unfair and disproportionate harms to vulnerable populations including increases in malaria in Africa and natural disasters in Central America, and the 2005 Chicago heat wave that affected primarily elderly African American men. He argues that those who generate the most emissions should bear at least some of the burdens, and willingness to accept this responsibility might be obtained by redefining climate change as a moral problem, he says, because ‘that something is the morally right thing to do is a powerful consideration in its favor. It may not always carry the day, but it cannot easily be ignored’ (Jamieson, 2008: 269–270).

Emissions *are* a moral problem, says Madison Powers (2014), because international, national and other policies that permit them to rise affect access to, and control over, energy, food and water, and because emissions raise ‘fundamental questions about the paths to economic development, poverty alleviation, and the capacity of individuals and nations to secure the basic requirements for decent human lives’. To reduce associated injustices, we need broad stakeholder consensus about the value of doing so. Public and policy dialog about scientific evidence, economic perspectives, security concerns, human rights and social justice is needed to attain this consensus, and these considerations must also be integrated into environmental and health law and governance (Wiley, 2010).

Bioethics often extends into, and should engage with, these issues, but only a handful of prominent bioethicists have done so. While emissions, climate change and connections between health and environment receive little attention, even in environmental and public health ethics, they are explicitly addressed in climate ethics literature—but this is seldom published in bioethics journals. From climate ethics, Henry Shue (2008) suggests that the wealthy ought to help rectify even unintentional damage from emissions because they profit the most from emissions, and that the poor are less accountable for emissions because their circumstances require them to prioritize the pursuit of food, water and goods essential to their survival over the protection of public goods. Distinguishing between subsistence and luxury emissions, Shue (2008) raises questions about what specific activities constitute subsistence needs, and contribute to subsistence emissions.

Values and ethics must be part of policy dialog because they underlie determinations about the extent to which nations should prioritize emissions reduction over other goods, and about what entities should contribute to reductions, James Garvey (2008) explains. He describes wealthy nations’ production of emissions as akin to stealing because ‘We have not just consumed a little more than the poor. We’ve taken a possible future from them and replaced it with something much worse … at the very least, we should begin to redress the balance by reducing our [own] emissions’ (Garvey, 2008: 73). We should do this now while the costs are still relatively low, says Nicholas Stern (2008), who recommends that nations agree to proportional targets and sanctions, and assess and report their successes in meeting those targets.

Peter Singer (2011) points out that negotiations about emissions reduction targets and policies must be built upon a global ethic, but that identifying this ethic is difficult because ‘causing imperceptible harm at a distance by the release of waste gases is a completely new form of harm, and so we lack any kind of instinctive inhibitions or emotional response against causing it. We have trouble seeing it as harm at all (237) … given the gravity of the risks … the level of protest against inaction has, to date, been quite small’ (217).

Potter’s global bioethics calls for interdisciplinary scientific and moral analyses to promote the present and future well-being of humanity (ten Have, 2012). Despite its focus on patient care, bioethics has the ability to highlight the benefits of emissions on one side and their harms to health and social justice on the other. Across disciplines and sectors, bioethics can help us see emissions as the serious harms that they are. We challenge readers to, at the very least, initiate dialog within their own institutions about the damages of emissions, the intrinsic value of nature and its extrinsic value to health and well-being.

### Nurturing Interdisciplinary Partnerships

The first bioethicists were physicians, philosophers, lawyers and nurses whose interdisciplinary collaborations addressed doctor–patient relationships, research ethics and end of life care (Callahan, 2012). Relatively few were then concerned with the reliance of health on nature and environmental resources, or the well-being of present and future populations. The global bioethics framework offers a way to document health impacts and other harms of emissions; assess their relative burdens in different places; advance and apply justice theories to policies and practices; and catalyze constructive dialog about the value of reducing emissions, perhaps partly by finding ways to meaningfully integrate them into medical and other curricula. Doing all this requires efforts to document, understand and respond meaningfully to emissions; and partnerships with health-care providers; mathematicians, computer modelers and economists; agricultural, atmospheric, biological, environmental, geographic, marine and veterinary scientists; industrial, political and policy leaders; behavioral and social scientists; and others.

One vehicle for such work is the One Health movement that reflects the convergence of human, animal and environmental health, and the need for integrated approaches to improve management of associated challenges (Gomez *et al.*, 2013). For example, the first transmission of West Nile virus in the Western Hemisphere occurred in 1999 when mosquitoes bit, infected and killed birds, horses and humans in New York: warm temperatures helped spread the virus which ultimately caused over 1000 deaths across the USA including, in 1999, 7 deaths, 10 cases of paralysis and 59 cases of meningoencephalitis among previously healthy people (Shomaker *et al.*, 2013). Documentation, understanding and management of this outbreak required interdisciplinary communication and partnerships. Bioethics might have complemented and extended their successes.

Specialist expertise in science, medicine, governance, policy, etc. permits progress in these realms but can hinder interdisciplinary work by impeding communications, conceptual understandings and opportunities, across specialties. This hindrance can be overcome by those interested in advancing human well-being. The Cambridge Program for Sustainability Leadership at Cambridge University, for example, engages business and health leaders in dialog about cost-effective means of improving sustainability. The Global Alliance for Assimilation of Information at Johns Hopkins University facilitates related dialog among scientific, health and security sectors. The Rock Ethics Institute at Penn State University, Centre for Sustainable Healthcare at Britain’s National Health Service and journals like ‘Nature Climate Change’ similarly show that interdisciplinary communication and partnership is feasible without discipline-specific jargon, dogma, methodologies and journals. Interdisciplinary work on emissions can help prevent further harms, and bioethics, with its interdisciplinary history, ought to sign on.

### Making Transitions

Rene Fox and Judith Swazey (2010: 280) describe how cultural influences in wealthy Western nations point bioethics toward autonomy and divert its attention from health, well-being and social justice. They call for ‘a collective will to undertake the intellectual work that we believe is needed to make the overarching conceptual framework and the ethos of the field more knowledgeably responsive to social and cultural context and diversity’. In addition to benefiting health and well-being for present and future populations, such work would enhance bioethics by broadening its appeal, balancing its emphasis on individuals with attention to populations, and shifting away from its dogmatic focus on individual autonomy.

This is a useful shift, says Angus Dawson (2010: 223), because ‘dependency is not a weakness but a fact of human life. Much of what we value in our lives arises from what we share together as social creatures. This fact is morally relevant and ought to be the foundation for the way we see bioethics. … How could we say anything interesting about animals if we are obsessed with autonomy? How do we explain the obligations that many of us feel towards those in need in far-off geographical regions … Can we really do what we like to an environment until our actions are banned?’ Grappling with these questions would enhance bioethics global relevance.

Jamieson (2008) posits that no one in 1900 would have imagined that development of the car would lead to interstate highways, air and noise pollution or lengthy commutes. He thus suggests that conventional morality is unable to assign accountability for such damages, and that doing so requires new conceptions of responsibility involving virtues like altruism (Jamieson, 2008). Once greatly admired, altruism is rarely visible in today’s leaders, but would equip them to better manage self interests that obscure the harms of emissions to themselves and others, and that obstruct actions to reduce emissions. (Global) bioethics invites reflection about how to nurture altruism and other virtues in leaders and policymakers, and how to generate public understanding of the economic and moral costs of failing to do so.

Bioethics is often seen as an umbrella over public health ethics, environmental ethics and other ethics involving living things. The relative isolation and applicability of these specialties to individual relationships restrains bioethics attention to them. Bioethics and its specialties have countless opportunities to explore ethics and policies within their own specialties and institutions. It is time to grasp these opportunities, address the evolving circumstances and values associated with globalization and expose policies that harm health.

## Conclusion

Our data support other evidence that emissions harm health, show that context affects the significance of a given harm in a given location and provide a foundation from which bioethics can initiate and inform public and policy dialog about priorities, strategies and associated values. Here, we highlight the generalizability of the data; unfair distribution of emissions-related benefits and burdens; need for interdisciplinary and comparative information with which to assess relative health burdens in different places, and respond sensitively to these differences; and need for interdisciplinary partnerships that embrace a global bioethics perspective.

We urge readers to reflect on connections between health, nature and values that might enhance care of our natural environment, and initiate dialog within their institutions about emissions, altruism and social justice. Bringing these concerns into its research and teaching will advance bioethics and facilitate interdisciplinary partnerships needed to understand and reduce global emissions. Imagine the benefits if even 5 per cent of bioethicists shifted their work from clinical dilemmas in wealthy settings to environmental conditions that threaten individual and collective health and well-being everywhere.
